# Factors Influencing Willingness to Continue Using Online Sports Videos: Expansion Based on ECT and TPB Theoretical Models

**DOI:** 10.3390/bs14060510

**Published:** 2024-06-20

**Authors:** Li Pan, Xinyi Pan, Xiaohong Mo, Tiansheng Xia

**Affiliations:** School of Art and Design and Guangdong International Center of Advanced Design, Guangdong University of Technology, Guangzhou 510090, China; panli@gdut.edu.cn (L.P.); 3219008024@mail2.gdut.edu.cn (X.P.); jackiemo@mail2.gdut.edu.cn (X.M.)

**Keywords:** online sports videos, theory of planned behavior, expectation-confirmation theory, social presence

## Abstract

Digital sports, also known as online sports, are a new form of sport that users have tried in recent years. Despite the rapid growth of online sports, the factors influencing users’ willingness to sustain their use are currently unknown. Based on the theory of planned behavior (TPB) and expectation-confirmation theory (ECT), this study empirically investigates the factors influencing the persistent use of online sports videos. Questionnaires were administered to participants. A total of 305 participants completed the questionnaire. Structural equation modeling showed that all hypotheses’ paths were significant, except for H11 and H12. The results indicated that perceived usefulness, expectation confirmation, and coach social presence had a significant positive effect on users’ satisfaction in using online sports videos. Moreover, satisfaction, behavioral attitudes, subjective norms, and perceived behavioral control had a significant positive effect on users’ willingness to consistently use online sports videos, with gender moderating the impact of satisfaction and behavioral attitudes on the willingness to consistently use. We discuss the practical implications and recommendations for applying this study’s findings.

## 1. Introduction

In an era when promoting health is paramount, physical exercise is becoming an increasingly indispensable part of people’s lives. Physical activity enhances physical qualities such as flexibility, balance, and strength [1], and also positively affects stress by increasing self-esteem and reducing anxiety and depression [2]. Participation in short- and long-term physical activity has both physical and psychological benefits [3]. It has always been the case that most people would choose offline venues to follow coaches for exercise. However, the advent of the digital age, especially since the major outbreak of COVID-19, has limited people’s outings and interpersonal interactions, leading to the closure of numerous sports and fitness venues [4]. Many people are turning to online fitness, transforming exercise and fitness into a digital experience. Subsequently, many products related to exercise and fitness have started to emerge, with mobile fitness apps being widely used to enhance people’s participation in physical activity [5]. Most fitness applications are equipped with video tutorials, specific needs training program development [6], exercise tracking, and social interactions. Exercising using video tutorials is the primary purpose of using fitness apps. However, most researchers have focused on fitness apps as a whole, while few have conducted in-depth studies on online sports videos [7,8]. Users favor online sports videos because of their convenience and autonomy, as well as their rich content and affordability. For example, figures like Genghong Liu and Pamela, prominent sports video bloggers with a high user focus in China, use short videos for exercise instruction. Previous research has found that the internet and social media platforms provide equal opportunities for each user to express and share, but they also lead to information overload and variable information quality [9], with wide variations in their usability and user experience. Conflicts between exercising and watching the videos may also negatively affect user experience [10]. In addition, there may be social interactions with others when users utilize online sports videos for fitness, but such social interactions are much weaker than in offline exercise contexts. Therefore, their impact on user experience and product usage intentions requires further investigation [11].

Exercising using online sports videos is part of an individual’s information and communications technology (ICT) usage behavior. The expectation-confirmation theory (ECT) is commonly used to explain usage behavior in various ICTs [12]. This theory elucidates the decision-making process users undergo when determining their consistent use of a product. The variables within the theoretical model may explain the factors influencing decisions regarding the ongoing use of online sports videos. By understanding the content of existing online sports videos, they can be broadly categorized into the leisure and fitness category as well as the exercise rehabilitation category based on the mode of exercise. Both of these may change in terms of the role that ECM variables have on them due to the difference in type. In the category of exercise rehabilitation, for example, it has been studied that YouTube sports videos can only provide partially reliable and moderate-quality information for rheumatoid arthritis patients. However, patients should be cautious not to rely directly on YouTube sports videos [13]. This indicates that patients with rheumatoid arthritis may have lower levels of expectation confirmation, satisfaction, and corresponding effects of perceived usefulness attitudes, which may lead to changes in the ECM when using online sports videos for rehabilitation. The subject of this study belongs to the leisure and fitness category of online sports videos, and it is also worth seeing whether the ECM produces changes when applied to this domain.

Similarly, the theory of planned behavior (TPB) also addresses decision-making behavior, focusing on users’ initial acceptance of a product rather than its continued use. This theory suggests that human behavior results from deliberate planning, and understanding the factors influencing this planning process can shed light on how people change their behavioral patterns. The theory of planned behavior model describes how three main variables, behavioral attitudes, subjective norms, and perceived behavioral control, affect the user’s intention to use a product, which in turn affects the actual behavior of the user’s use. The degree to which these factors are high or low will have an impact on an individual’s intention, i.e., an individual’s intention for the behavior is higher when they have a more positive attitude, more encouragement and support from others, and more confidence in controlling the influencing factors [14]. Additionally, scholars have conducted research on whether these three variables continue to affect the willingness to sustain use after influencing actual use behavior. Jeng et al.’s study demonstrated that attitudes, subjective norms, and perceived behavioral control all influence older adults’ intentions to continue in virtual reality leisure activities [15]. Hopkins et al. used the TPB to examine the intentions of adolescent female athletes to continue participating in sports, and the results indicated that these three variables were similarly validated for their effects on intentions to continue participating in sports [16]. All of the above studies verified that the independent variables in the TPB are the influencing factors that affect users’ continued participation in sports and that the purpose of users’ use of online sports videos is to engage in sports, so we can speculate that there is appropriateness in utilizing the variables in the TPB model to explain the factors that affect the decision to continue using online sports videos. This is also consistent with the comparative findings of past studies that TPB has strong explanatory power for both use and persistence [17]. It can be understood that if users have a positive attitude towards using online sports videos, feel that they have environmental conditions and operational capabilities to easily access and use online sports videos, and, at the same time, receive encouragement and approval from their family and friends, they will have a stronger intention to continue using them.

Taken together, the use of online sports videos for at-home exercise has become a trend that continues to grow in popularity, making it one of the most important ways for people to be physically active and stay healthy. However, it is unclear which factors influence users’ willingness to continue using online sports videos. This study aims to bridge this gap by investigating the factors affecting users continued use of online sports videos from a theoretical perspective, which is conducive to providing theoretical guidance for related work on online sports videos, providing users with a convenient and comfortable exercise channel, enhancing their use of retention rates, and helping to improve public health. Considering the characteristics of the ECM and TPB, this study incorporates the TPB as a supplement to the ECM to comprehensively understand users’ selection and consistent use of online sports videos for exercise practice. Previous research—such as Li’s (2022) integration of the TPB and ECM models to investigate the factors influencing Chinese students’ persistent willingness to learn online—has demonstrated the feasibility of this approach [18]. Online sports video workouts, while distinct from traditional online learning, are akin to distance learning. Thus, insights from relevant studies on distance learning are valuable for this study. Therefore, this study selected an integrated model combining the ECM and TPB to investigate the factors and mechanisms influencing users’ persistent willingness to use online sports videos. The goal of the study is to explore the factors influencing the continued use of online sports videos in order to guide online sports video developers in platform optimization and user experience enhancement. Specifically, the study targets young people who have the need to use online sports videos for exercise, and the intervening factors involve behavioral attitudes, subjective norms, and other original variables in the theoretical mode. By comparing the differences in different variables, we can identify the factors that have a significant impact on the intention to continue using online sports videos and the strategies for service enhancement.

## 2. Research Modeling

### 2.1. Expectation-Confirmation Theory

**Figure 1:**
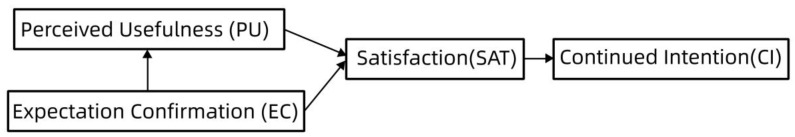
Expectation-confirmation theoretical model.

Expectation-confirmation theory comprises four variables: expectation confirmation (EC), perceived usefulness (PU), satisfaction of users (SAT), and continued intention of users (CI). These mainly describe how consumers assess their satisfaction with a product or service based on a comparison between their pre-purchase expectations and the product’s performance during usage, influencing their decision to continue using it [19]. This theory suggests that consumers have an initial expectation upon purchasing a product, and after experiencing it over time, they form new perceptions based on their experience. Subsequently, they compare their new perceptions with their pre-purchase expectations, the results of which affect the user’s assessment of the perceived usefulness of the product, which in turn affects their intention to continue using it. Expectation confirmation and perceived usefulness also influence satisfaction, further shaping users’ intentions to continue using products. Previous studies have predominantly focused on information technology, such as e-learning [20,21]. Choi et al. applied the ECM to analyze the formation of user habits after using a short mobile video platform [22]. Joo and Choi (2016) examined the factors influencing the willingness to continue using online library resources based on an extended ECM [23]. Other scholars have explored the persistent intention of online users to watch AI anchor reports from an ECM perspective [24]. However, there is currently limited research using the ECM to explore users’ intentions to use online exercise/fitness videos. Considering that online sports videos are pedagogical in nature and belong to the category of online learning videos, we refer to the literature related to online learning. Specifically, several researchers have used the ECM to study the continuous use of online learning services [25,26,27,28]. Figure 1 illustrates the main framework of this model.

In this study, we aim to confirm the alignment between users’ expected and actual experiences. The degree of expectation confirmation affects users’ perceived usefulness of a product/service and subsequent satisfaction with the product/service. Based on the objectives of our study, we propose the following hypotheses:

**H1.**  *Users’ level of expectation confirmation positively correlates with their perceived usefulness of online sports videos;*

**H2.**  *The perceived usefulness of online sports videos positively affects user satisfaction.*

According to the ECM, user satisfaction predicts continuation intent, and when user expectations are met, satisfaction increases [29,30,31,32]. For example, satisfaction with the online learning platform MOOC rises when expectations are fulfilled. We propose the following hypothesis:

**H3.**  *The degree to which users’ expectations are confirmed positively relates to their satisfaction with online sports videos.*

Many studies on information systems’ continuation intention have demonstrated the applicability of the ECM [33,34,35,36]. In this model, satisfaction significantly influences users’ intention to continue using a product or service. Accordingly, we propose the following hypothesis:

**H4.**  *Users’ satisfaction with online sports videos positively correlates with their intention to continue usage.*

### 2.2. Theory of Planned Behavior

**Figure 2:**
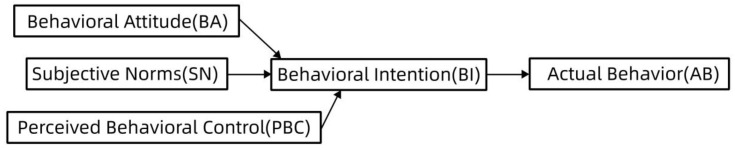
Theoretical model of planned behavior.

The theory of planned behavior, proposed by Ajzen [37,38], has been extensively used to explore rational human decision-making behavior [39]. This theory comprises five key variables: behavioral attitudes (BAs), subjective norms (SNs), perceived behavioral control (PBC), behavioral intentions (BIs), and actual behavior (AB). Behavioral attitudes, subjective norms, and perceived behavioral control jointly influence users’ behavioral intentions, which in turn affect actual behavior. In recent years, scholars have applied the TPB across various fields of research, such as new technology and education, to explain the elements of personal behavioral influences in different environments. Luo and Wei used this model to investigate individuals’ intentions to use shared self-driving cars [40]. Luo et al. integrated the TPB with the technology acceptance model (TAM) to explore students’ e-book reading intentions [41]. Other researchers have combined technology acceptance and planned behavior theoretical models to investigate college students’ continued willingness to use online learning platforms [42]. Figure 2 illustrates this model.

When applied to new research areas, the TPB often requires adaptation or extension to fit a particular existing technology or system [43,44]. We applied the TPB to explore the factors influencing users’ choice to utilize online sports videos.

Considering the behavioral attitude factor first, the TPB suggests that an individual’s behavioral attitude is positively related to their behavioral intention. College students’ intent to use e-learning was found to be favorably connected with their behavioral attitude [45], and students’ behavioral intentions were found to be positively impacted by their attitude toward MOOCs [46]. Given this background, it is reasonable to expect that users’ continued willingness to engage with online sports videos may be influenced by their attitudes toward them. Simultaneously, we drew upon the TAM, a theoretical framework developed by Davis [47], which explains why people accept or reject new technologies [48]. In the TAM, perceived usefulness is also hypothesized to have a positive impact on attitudes, subsequently influencing behavioral intentions [49]. Based on the underlying TPB, subjective norms and perceived behavioral control similarly influence the intention to consistently use a product or service. Thus, these hypotheses are proposed:

**H5.**  *Users with positive attitudes will be more likely to consistently exercise using online sports videos;*

**H6.**  *Perceived usefulness significantly and positively influences users’ attitudes toward using online sports videos;*

**H7.**  *Subjective norms positively influence the willingness to consistently use online sports videos for exercise;*

**H8.**  *Perceived behavioral control positively influences users’ willingness to consistently use online sports videos for exercise.*

### 2.3. Social Presence: Coach and Peer Social Presence

Social presence theory was first developed by Short, Williams, and Christie [50]. Social presence was originally defined as the degree to which one person perceives another as salient or “there” when interacting in a computer-mediated communication environment. Subsequent researchers have further refined this definition to denote “the extent to which a person is perceived as a real person in mediated communication” [51]. Scholars in online research use the social presence theory to describe the dynamics of social interactions in online learning environments [52]. Given its focus on human interaction and feelings of sociability, social presence is often considered a crucial factor in studies on how online learners’ relationships with teachers and other students are perceived. Social presence has been shown to affect students’ motivation, course and instructor satisfaction, and the retention rates of online courses [53]. In our study, we considered two main variables, course satisfaction and retention, that is, user satisfaction with online sports videos and willingness to continue using them.

Several studies across various fields have found that social presence affects student satisfaction [54,55,56,57,58]. However, others have found the opposite. For instance, Guo et al. studied online learning and concluded that learners perceived the presence of others as having little or no impact on them. In particular, many Chinese students do not like to discuss with others while learning and prefer to achieve their learning goals independently [59]. Therefore, when studying the new topic of online sports videos, it is necessary to reintroduce the factor of social presence into the research scope. This is because online sports videos differ from conventional online learning materials, and the results of previous studies may not be applicable. In the fields of teaching and learning, social presence refers to the interaction and perception among students and between students and teachers [60,61]. Here, we refer to the existing literature and categorize social presence into coach and peer social presence. Coach social presence refers to students’ perception of the coach. Peer social presence pertains to students’ awareness of each other, communication comfort, and perceptions of fellow students [62]. Based on the effect of social presence on satisfaction, the following hypotheses are proposed:

**H9.**  *Coach social presence positively influences user satisfaction in using online sports videos;*

**H10.**  *Peer social presence positively influences user satisfaction in using online sports videos.*

Social presence has also been shown to positively influence willingness to persist on an online learning platform, such as MOOC. Chen verified that learner–teacher interactions and learner–learner interactions in a MOOC have a positive impact on MOOCs. These interactions trigger both instructional and social presence, which jointly enhance the intention to persist in using the MOOC [63]. Therefore, we hypothesize the following:

**H11.**  *Coach social presence positively influences users’ intention to consistently use online sports videos;*

**H12.**  *Peer social presence positively influences users’ intention to consistently use online sports videos.*

**Figure 3:**
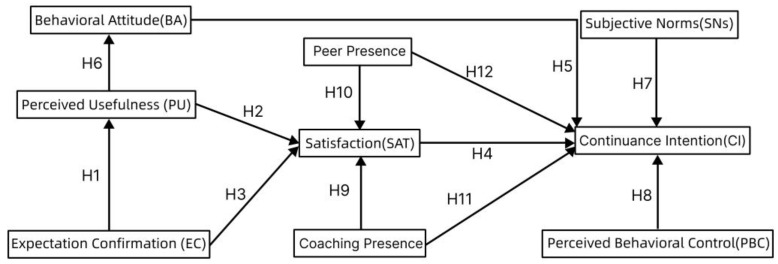
Research framework and hypotheses.

The framework and hypotheses of this study are illustrated in Figure 3. Expanding on the ECM and TPB, the key variables identified for the framework of this study include expectation confirmation, perceived usefulness, behavioral attitudes, satisfaction, subjective norms, perceived behavioral control, coach social presence, peer social presence, and willingness to sustain use.

## 3. Methodology

### 3.1. Participants

Three hundred and thirty-eight volunteers participated in the survey. A total of 305 valid questionnaires were received after the unqualified ones were eliminated. There were 169 females (65.90%) and 104 men (34.10%) among the participants, and they were between the ages of 18–25 (mean age: 22.34, standard deviation: 2.78). The majority of the respondents reported having experience using online sports videos for exercise and fitness (87.54%). This study was approved before being carried out.

### 3.2. Measurement Development

A questionnaire, developed through a series of steps, was used to measure the relevant research variables. First, the literature on online learning was reviewed, and some questions were modified to suit the current topic of online sports videos [64,65,66,67,68]. Thereafter, three experts in questionnaire design were invited to review and advise on the questionnaire items. The final questionnaire was finalized by refining the details based on the experts’ advice. The questionnaire included nine variables involved in the research model. “Willingness to continue to use” contained two items, while “coach social presence” and “peer social presence” each comprised four items. The remaining variables contained three items each, which were assessed on a five-point Likert scale. A complete list of the items and their sources is provided in the Appendix A.

### 3.3. Data Collection

To ensure the validity of the information, a pre-survey was conducted before the release of the official questionnaire with a sample size of 60. The collected data samples were tested for reliability and validity to ensure the reliability of the results of the subsequent data analyses. The results were deemed satisfactory before proceeding to the formal questionnaire data collection. The formal questionnaire was administered to undergraduate and postgraduate students. It was created using “Questionnaire Star” “https://www.wjx.cn/ (accessed on 1 November 2023)”, and the questionnaire link was distributed to respondents through WeChat friends, group chats, and friend circles. It comprised three sections: an introduction, demographic attributes, and measurement items. The first section provided an introductory statement, while the demographic attributes section collected information on gender, age, and online sports video usage. The measurement items section assessed nine variables involved in the model. The questionnaire data were collected from 1 November to 10 November 2023.

### 3.4. Reliability and Validity Analysis

The samples were tested for convergent validity, and the factor loadings were almost always greater than 0.6, indicating a good model fit. Subsequently, the CR and AVE were calculated. CR represents construct reliability, which assesses whether the items in the test question consistently explain their corresponding variables. The AVE is the squared error-extracted value, which reflects whether the measured question items are consistent within each variable. In this study, most variables exhibited CR values surpassing 0.7 (except for behavioral attitudes and willingness to continue to use), while some AVE values were below 0.5. However, it has been established and confirmed that an AVE value exceeding 0.36 is considered acceptable [69,70]. Therefore, the overall convergent validity reached the standard, and the discriminant validity test could be continued. The reliability and convergent validity of the results are presented in Table 1.

Finally, Pearson’s correlation analysis of these variables showed significant pairwise correlations (Table 2). The values on the diagonal of the table represent the square roots of the calculated AVE values obtained using AMOS 23.0. In validity analysis, discriminant validity requires that the square root of the AVE value of each variable be greater than the correlation coefficient between the variables. Table 2 shows the results of the discriminant validity test for the sample. It is evident that the correlation coefficients between the variables were less than the square root of the AVE values on the diagonal, indicating good discriminant validity.

### 3.5. Model Testing

Based on the online sports video user behavior model, a structural equation model was constructed using AMOS 23.0. Before verifying the hypotheses, the model’s fit was assessed using the goodness-of-fit index, which is a prerequisite for validating the results of the subsequent hypotheses tests. By referring to the previous literature, this study applied the following metrics: ratio of χ^2^ to its degrees of freedom (χ^2^/df), goodness-of-fit index (GFI), comparative fit index (CFI), Tucker–Lewis index (TLI), and the root mean square error of approximation (RMSEA). During the debugging process, the model was corrected based on the suggested modification index (MI). In the final structural equation model, χ^2^/df = 2.116, GFI = 0.871, CFI = 0.902, TLI = 0.901, and RMSEA = 0.062. The model fit was good and within acceptable limits.

The final result showed that the paths of all the hypotheses were significant except H11 and H12. H1, H2, H6, and H7 were significant at the 0.001 level, while H3, H4, H8, and H9 were significant at the 0.01 level, and H5 was significant at the 0.05 level. It is noteworthy that the path coefficient of H10 (β = −0.436, *p* < 0.01) was negative, indicating that peer social presence negatively affects users’ satisfaction with online sports videos. The results of all the tests are summarized in Table 3.

With the inclusion of the gender-moderated variables, the results showed a significant difference between genders (*p* < 0.01). As shown in Table 4, H1, H3, H5, H6, H9, and H10 garnered support from the male respondents, while all hypotheses except H5, H7, and H12 garnered support from the female respondents. Gender had a moderating effect on H4 and H5.

In addition, participation in this questionnaire included users who had used online sports videos as well as those who had not. Therefore, it was possible to use the experience of use as a moderating variable, and the results showed that there was a significant difference between these two cases (*p* < 0.05). As shown in Table 5, H1–H10 supported users with usage experience, while all hypotheses except H7, H8, and H12 supported users without usage experience. The presence or absence of usage experience had a moderating effect on H9, H10, and H11.

## 4. Discussion

This study combined the ECM and TPB while introducing the new variable social presence with the aim of exploring the factors influencing the continued use of online sports videos to guide the developers of online sports videos for platform optimization and user experience enhancement. We examined an integrated framework using structural equation modeling and obtained some meaningful results. Of interest is that neither coaching nor peer social presence significantly affects users’ intention to consistently use online sports videos, while peer social presence has a significant negative effect on user satisfaction. Specific discussions on the results are presented below.

### 4.1. Theoretical Contributions

This study contributes in several ways. First, it integrates two theories, the TPB and ECT, to explore online sports videos. As an emerging online method, many studies on online learning videos have focused on technology acceptance. This study extends previous research by focusing on the factors that influence users’ choices and whether they will continue to use online exercise videos. Second, this study incorporates social presence, an important influencing factor in online learning, into its model. Through an in-depth literature review, we addressed the previous limitation of treating social presence as a one-dimensional structure. Instead, we categorized it into two dimensions: coach and peer social presence. They were then introduced as independent variables into the newly constructed model, aiming to explore the specific roles played by the coach and peer social presence in online exercise scenarios. This study also determined whether there was a significant effect of the two on online exercisers and provided a comprehensive understanding of how coaches and peers influence learning satisfaction and sustained intentions. These can help us better understand the role of social presence in online sports videos.

### 4.2. Key Findings

Our result confirms the applicability of the ECM to the context of online sports videos, yielding valuable conclusions. The degree of expectation confirmation has a direct effect on perceived usefulness. Furthermore, satisfaction mediates the effects of the degree of expectation confirmation and perceived usefulness on the willingness to continue using online sports videos. Additionally, perceived usefulness also mediates the relationship between expectation confirmation and behavioral attitudes. Hypotheses related to the TPB were also tested, revealing that both subjective norms and perceived behavioral control have a direct effect on the willingness to continue using online sports videos. In addition, the mediating role of behavioral attitudes in the process of perceived usefulness on the willingness to continue using online sports videos was confirmed. Overall, the ECT and TPB integration frameworks identified in this study were validated.

This study introduced coach and peer social presence as direct factors assumed to influence satisfaction and willingness to continue using online sports videos. Regarding the question of whether social presence improves student satisfaction, most of the previous findings reported a positive effect of social presence on satisfaction, whereas a few reported a negative effect. The results of this study revealed that the social presence of coaches has a significantly positive effect on user satisfaction, whereas the social presence of peers has a significantly negative effect on user satisfaction. This suggests that a coach’s social presence fosters a comfortable subjective feeling among users, which is conducive to increasing satisfaction. Conversely, peer social presence engenders a subjective feeling of discomfort in users during exercise sessions with online sports videos, leading to reduced satisfaction.

A possible reason for this finding is that, unlike other online learning videos, users of online sports videos primarily engage in imitating and practicing fitness movements with the instructor while watching. It is well known that it is easy to experience physical exhaustion when exercising, leading to a decline in motivation. However, coaches can often play a great role in encouraging students to persist in completing exercise tasks by interacting with them in the form of gestures and facial expressions. Therefore, it can be interpreted that the social presence of coaches can directly enhance user satisfaction. Random interviews with questionnaire respondents and observations of interactions among video viewers in online sports videos revealed that video viewers usually communicate through pop-ups, through which they record their fitness activities and share their workout results. This may create a sense of anxiety for users who have just started exercising or who are eager to obtain their workout results. Additionally, because users must perform movement exercises while watching the video and cannot operate the playback device’s screen simultaneously, interaction with online peers and controlling movements both consume cognitive resources, leading to a certain conflict.

Additionally, previous studies have found that social presence enhances the intention to persist in online learning, often through a multidimensional approach. However, the present study yielded different results, showing that neither the social presence of the coach nor peers significantly affected users’ intentions to persist with online sports videos. This may be due to the fact that, with reference to offline exercise, most people choose to exercise alone once they enter a state of physical activity, thus avoiding distractions and physical exertion caused by communicating and interacting with others. Distractions during exercise are more dangerous than those in other learning situations and can lead to physical injuries. It is, therefore, understandable that, for online exercisers, once they become familiar with the online exercise approach, the social presence of the coach and peers is not significant enough to affect their willingness to continue using online sports videos for exercise. Our results differ from those of previous studies and demonstrate the specificity of online sports videos compared with other online instructional videos.

After including gender as a moderating variable, a significant difference between genders was observed. While gender has been previously included as a moderating variable in the field of online fitness based on structural equation modeling [71], it primarily focused on empirical research concerning the factors influencing users’ behavior in using fitness software. Our result is consistent with the previous findings, which also affirms the moderating role of gender. This finding highlights the variations in attitudes and satisfaction with online sports videos across genders, influencing continued usage intentions and leading video operators to tailor and personalize their services and content to gender-specific users. However, the specific reasons for this finding remain unexplored, and future research should continue to investigate gender-based differences in the use of online sports videos.

### 4.3. Practical Implications

Our findings hold several practical implications. First, it confirms that coaches’ social presence positively influences satisfaction with online sports, suggesting that coach–participant interaction is necessary in an online sports scenario. Therefore, we suggest that operators of online sports video platforms establish a more intimate and immersive online environment for athletes to engage with coaches. This may include dedicated dialogue zones and interactive tools for feedback. This may help to recreate a sense of interaction with the trainer in actual offline practice scenarios, thus improving the experience and satisfaction of online exercisers. Second, in addition to platform operators, we suggest that online sports video instructors be more interactive when teaching to enhance learner immersion and intimacy. At the same time, the results of this study’s research model analysis indicate the feasibility of a mechanism model for influencing online sports video satisfaction and continuance intentions based on the original ECT and TPB theories. This implies that producers of online sports videos and platform providers can utilize this model as a reference and adopt appropriate facilities in their practice.

### 4.4. Limitations and Future Research

This study also has several limitations. First, during the questionnaire collection stage, the main participants consisted of undergraduate and postgraduate students from a single university. Although college and postgraduate students are the main user groups of online sports videos, the actual user base covers a wide range of ages and occupations, potentially affecting the generalizability of the results. Future research could address this limitation by incorporating data from multiple online sports video provider platforms to gain a more comprehensive understanding of the impact of different groups on the continued use of online sports videos.

Second, this study proposes that the social presence of coaches and peers is an important antecedent of satisfaction and intention to continue using online exercise videos. The results show a positive correlation between the coaches’ social presence and user satisfaction. We also tried to explain the reasons for the non-significance of the other hypotheses related to social presence. However, their explanatory power is yet to be verified and improved, and the final addition of gender moderation has not yet been explained by consulting reasonable theories. Subsequent in-depth studies should be conducted mainly on social presence and gender.

Third, this study’s reliance on various platforms offering online sports videos introduces potential external factors that may have influenced the results. Although we used an expert-vetted questionnaire, experience with online sports videos is highly subjective. Respondents’ answers may have been influenced by individual subjectivity and differences in the platforms they used. Therefore, future research should explore the incorporation of objective measures such as physiological responses or behavioral observations to more comprehensively assess the factors influencing online sports videos. For example, when studying online sports videos, factors such as individual preferences and platform differences may have influenced the results.

Finally, it is also important to note that even in online sports videos, the descriptive terminology and user experience vary depending on how the viewer participates. The two main types of viewer participation are direct participation and indirect participation. Direct participation refers to the actual exercise workout performed by the viewer following the instructions and directions in the video. In this approach, the user’s need for real-time feedback and interaction may be higher, and their experience may be directly affected by the quality of the video content, the instructor’s coaching style, the interactivity, as well as the clarity and sound of the video. Indirect engagement, on the other hand, refers to viewers watching videos but not performing actual workouts, such as watching recorded videos of fitness classes, in which case users pay more attention to the informativeness of the videos and the professionalism of the content. This study focuses on the usage scenarios of direct engagement. In the future, we can try to investigate how viewers’ engagement styles affect their expectations and experiences, and the differences between the two are likely to have important implications for platform optimization and user experience enhancement strategies.

## 5. Conclusions

This study expands upon the theories of the ECM and TPB by investigating the factors influencing users’ utilization and persistence with online sports videos. The majority of hypotheses were valid, except H10, H11, and H12. First, we found that the variables of perceived usefulness, expectation confirmation, and the social presence of coaches had a significant positive effect on user satisfaction with online sports videos. Second, satisfaction, behavioral attitudes, subjective norms, and behavioral control had a significant positive effect on users’ willingness to continue using online sports videos. Notably, we found that gender played a moderating role in the process of satisfaction and behavioral attitudes on the intention to continue using online sports videos. Meanwhile, our findings suggest that the degree of expectation confirmation positively affects perceived usefulness, whereas behavioral attitudes mediate the effect of perceived usefulness on the intention to continue using. Although our study revealed that multiple factors positively influence user satisfaction with online sports videos, peer social presence is a special case. Our results showed that the presence of peers negatively affects user satisfaction when online exercise videos are used for fitness.

In summary, this study focused on the factors influencing the continuous use of online sports videos and provided valuable insights and practical suggestions based on these findings. We expect the findings of this study to provide online sports video providers and video broadcasting platform operators with more effective video design ideas and operation strategies to enhance user retention and increase users’ willingness to continue using online sports videos.

## Figures and Tables

**Table 1:** Data on the indicators of confidence and convergent validity.

Construct	Item	Factor Loading	Cronbach’ Alpha	CR	AVE
PU	PU1	0.721	0.759	0.754	0.505
	PU2	0.671			
	PU3	0.739			
EC	EC1	0.663	0.711	0.717	0.458
	EC2	0.664			
	EC3	0.703			
BA	BA1	0.681	0.714	0.693	0.430
	BA2	0.710			
	BA3	0.570			
SAT	SAT1	0.759	0.732	0.720	0.464
	SAT2	0.669			
	SAT3	0.608			
SNs	SNs1	0.802	0.772	0.755	0.536
	SNs2	0.718			
	SNs3	0.670			
PBC	PBC1	0.685	0.704	0.705	0.444
	PBC2	0.612			
	PBC3	0.699			
CI	CI1	0.746	0.716	0.642	0.474
	CI2	0.626			
COA	COA1	0.717	0.814	0.808	0.512
	COA2	0.722			
	COA3	0.737			
	COA4	0.687			
COM	COM1	0.816	0.841	0.841	0.571
	COM2	0.742			
	COM3	0.693			
	COM4	0.767			

Note: PU = perceived usefulness, EC = expected confirmation, BA = behavioral attitude, SAT = satisfaction, SNs = subjective norms, PBC = perceived behavioral control, CI = continuance intention, COA = coach social existence, COM = companion social existence.

**Table 2:** Results of the sample differentiation validity tests.

	1	2	3	4	5	6	7	8	9
1. PU	0.711								
2. EC	0.445 **	0.673							
3. BA	0.416 **	0.359 **	0.656						
4. SAT	0.397 **	0.365 **	0.322 **	0.681					
5. SNs	0.420 **	0.332 **	0.346 **	0.369 **	0.732				
6. PBC	0.411 **	0.396 **	0.328 **	0.307 **	0.396 **	0.666			
7. CI	0.488 **	0.469 **	0.421 **	0.396 **	0.428 **	0.506 **	0.689		
8. COA	0.232 **	0.212 **	0.314 **	0.258 **	0.262 **	0.198 **	0.248 **	0.716	
9. COM	0.187 **	0.213 **	0.262 **	0.194 **	0.226 **	0.158 **	0.218 **	0.529 **	0.756

Note: ** *p* < 0.01.

**Table 3:** Summary of the results of the hypothesis testing.

Hypothesis (n = 305)	Unstd.	S.E.	C.R.	*p*	Std.	Remark
H1 EC → PU	0.672	0.077	8.752	<0.001 ***	0.814	Supported
H2 PU → SAT	0.515	0.152	3.395	<0.001 ***	0.454	Supported
H3 EC → SAT	0.422	0.132	3.209	0.001 **	0.451	Supported
H4 SAT → CI	387	0.129	3.009	0.003 **	0.422	Supported
H5 BA → CI	0.505	0.143	3.531	<0.001 ***	0.476	Supported
H6 PU → BA	0.904	0.089	10.145	<0.001 ***	0.923	Supported
H7 SN → CI	0.169	0.073	2.314	0.021 *	0.223	Supported
H8 PBC → CI	0.250	0.078	3.193	0.001 **	0.281	Supported
H9 COA → SAT	0.388	0.129	3.007	0.003 **	0.490	Supported
H10 COM → SAT	−0.299	0.110	−2.708	0.007 **	−0.436	Not Supported
H11 COA → CI	−0.148	0.162	−0.915	0.360	−0.204	Not Supported
H12 COM → CI	0.093	0.119	0.782	0.434	0.148	Not Supported

Note: * *p* < 0.05; ** *p* < 0.01; *** *p* < 0.001.

**Table 4:** Results of the gender-moderated effects.

Hypothesis	Path	Male Results	Female Results
H1	EC → PU	Supported	Supported
H2	PU → SAT	Not Supported	Supported
H3	EC → SAT	Supported	Supported
H4	SAT → CI	Not Supported	Supported
H5	BA → CI	Supported	Not Supported
H6	PU → BA	Supported	Supported
H7	SN → CI	Not Supported	Not Supported
H8	PBC → CI	Not Supported	Supported
H9	COA → SAT	Supported	Supported
H10	COM → SAT	Supported	Supported
H11	COA → CI	Not Supported	Supported
H12	COM → CI	Not Supported	Not Supported

**Table 5:** Results of the usage experience-moderated effects.

Hypothesis	Path	Have Experience	Have No Experience
H1	EC → PU	Supported	Supported
H2	PU → SAT	Supported	Supported
H3	EC → SAT	Supported	Supported
H4	SAT → CI	Supported	Supported
H5	BA → CI	Supported	Supported
H6	PU → BA	Supported	Supported
H7	SN → CI	Supported	Not Supported
H8	PBC → CI	Supported	Not Supported
H9	COA → SAT	Supported	Supported
H10	COM → SAT	Supported	Supported
H11	COA → CI	Not Supported	Supported
H12	COM → CI	Not Supported	Not Supported

## Data Availability

The data presented in this study are available upon request from the corresponding author.

## Appendix A

1. Perceived Usefulness (PU)	1. I have better sports performance when using online sports videos
	2. I have high exercise efficiency when using online sports videos
	3. I find online sports videos useful
2. Behavioral Attitude (BA)	1. I think using online sports videos is a good option
	2. I like to exercise using online sports videos
	3. I think it’s feasible to use online sports videos for exercise
3. Subjective Norms (SNs)	1. People who are important to me support me in exercising using online sports videos
	2. People who influence me think I should exercise using online sports videos
	3. People whose opinions I value think I should use online sports videos for exercise
4. Perceived Behavioral Control (PBC)	1. I will have the necessary resources, time and opportunity to use online sports videos
	2. Whether or not I exercise using online sports videos is entirely up to me
	3. Exercising with online sports videos is completely within my control
5. Expected Confirmation (EC)	1. My experience with online sports videos has been more than I expected
	2. The level of service provided by the online sports video was better than I expected
	3. Online sports videos can be catered for beyond my requirements
6. Satisfaction (SAT)	1. I’m happy with the performance of the online sports videos
	2. I’m happy with my experience of exercising using online sports videos
	3. My decision to use online sports videos for exercise was wise
7. Continuance Intention (CI)	1. I will continue to use online sports videos for exercise in the future
	2. I highly recommend it to others
8. Coach Social Existence (COA)	When I interact with my coach via video comments/likes etc……
	1. Feeling like we’re together
	2. I felt that the coach was interacting with me
	3. I feel like the coach knows I’m there
	4. The presence of a coach was obvious to me
	5. I felt comfortable talking to the coach
9. Companion Social Existence (COM)	When I interact with other viewers via comments/pop-ups etc……
	1. Feeling like we’re together
	2. I feel like the rest of the audience is interacting with me
	3. I think the rest of the audience is aware of my existence
	4. The presence of other viewers is obvious to me
	5. I feel comfortable interacting with them
